# Exploring the pathways between green space and blue space, and cognitive function in later life: a mediation analysis in Northern Ireland

**DOI:** 10.1136/bmjph-2025-003080

**Published:** 2026-10-07

**Authors:** Ruoyu Wang, Joanna McHugh Power, Hüseyin Küçükali, Ione Avila-Palencia, Shay Mullineaux, Jennifer McKinley, Frank Kee, Bernadette McGuinness, Ruth F Hunter

**Affiliations:** 1 Centre for Public Health, Queen's University Belfast, Belfast, Northern Ireland, UK; 2 NOVA Information Management School (NOVA IMS), Universidade NOVA de Lisboa, Campus de Campolide, Lisboa, Portugal; 3 Department of Psychology, Maynooth University, Co. Kildare, Ireland; 4 Research Center for Healthcare Systems and Policies, Istanbul Medipol University, Istanbul, Türkiye; 5 Institute for Risk Assessment Sciences, Utrecht University, Utrecht, The Netherlands; 6 Department of Geography, School of Natural and Built Environment, Queen’s University Belfast, Belfast, Northern Ireland, UK

**Keywords:** Epidemiology, Environmental Exposure, Age Factors, Community Health

## Abstract

**Introduction:**

The pathways by which green space and blue space exposure influence cognitive function are unclear.

**Methods:**

To investigate the association between green space and blue space exposure and cognitive function and potential mediating pathways through which this association may act, we used multilevel structural equation models and longitudinal data from Waves 1 and 2 of the Northern Ireland Cohort for the Longitudinal Study of Ageing study. Cognitive function was assessed by the Mini-Mental State Examination (MMSE). We assessed green space and blue space exposure using: (1) NDVI and NDWI (Normalised Difference Vegetation/Water Index); (2) accessibility of green space and blue space (distance to the nearest parks/coastlines); and (3) availability of green space and blue space (the proportion of vegetation/water bodies). Potential mediators included: (2) moderate-to-vigorous physical activity (MVPA); (2) walking; (3) social contact; (4) social support and (5) fine particulate matter with a diameter of 2.5 µm or less.

**Results:**

First, MVPA mediates the association between NDVI (Coef.=0.0017; SE=0.0008), NDWI (0.0035; SE=0.0015), accessibility of green space (Coef.=−0.0015; SE=0.0007) and MMSE scores. Second, social contact also mediates the association between availability of green space (Coef.=0.0023; SE=0.0009), accessibility of blue space (Coef.=−0.0009; SE=0.0004) and MMSE scores.

**Conclusion:**

Our findings suggested that green space and blue space exposure are important predictors of cognitive function in later life and that they may exert their effect via multiple pathways. Green space and blue space exposure may contribute to healthy ageing.

**WHAT IS ALREADY KNOWN ON THIS TOPIC**

**WHAT THIS STUDY ADDS**

- This is the first cohort study exploring the pathways between blue space exposure and cognitive function in older adults.
- Our study found that all green space metrics, Normalised Difference Water Index and accessibility of blue space influence cognitive function mainly through moderate-to-vigorous physical activity (MVPA) and social contact.
- There is no evidence that the availability of blue space is associated with cognitive function.

**HOW THIS STUDY MIGHT AFFECT RESEARCH, PRACTICE OR POLICY**

- Our findings suggest that higher levels of green space and blue space exposure may contribute to better cognitive function in later life, mainly through promoting MVPA and social contact.
- Promoting the provision of green space and blue space may be a strategy to contribute to healthy ageing.

## Introduction

 The proportion of the ageing population around the world will increase from about 12% to 22% (2015–2050).^1^ In Northern Ireland (NI), it is predicted that 20.1% of the population will be over 65 by mid-2028.^2^ It has been well documented in previous studies that cognitive function changes with the process of ageing.^3–7^ With concomitant growth in dementia rates worldwide,^8^ it is estimated that the ageing population living with dementia in NI is over 20 000 currently, and it will double by 2040.^9^ Therefore, it is important to understand the determinants of cognitive function and dementia among older adults both in NI and globally.^10^

Natural environments such as green space and blue space (eg, vegetation and water bodies) are modifiable through urban planning policy and have multiple health impacts.^11–16^ Although evidence is increasing regarding the positive association between natural environments and cognitive function,^17–21^ existing literature focuses mainly only on green space^22–25^ or on children and young adults.^26,27^ Some studies observed a positive relationship between blue space and mental health,^28,29^ but it is still unclear whether there are health impacts of blue space on the cognitive function of older adults. For example, regarding the health benefits of natural environments, the Stress Reduction Theory is more associated with mental health benefits, while the Attention Restoration Theory (ART) is more linked to improvements in cognitive function.^19^ Therefore, the mechanisms linking natural environments to cognitive function need to be further investigated. Also, since older adults may be less mobile and spend more time within their neighbourhood, green space and blue space near their residential location may be more important compared with young adults.^30^

Recent reviews have documented three potential pathways linking green space and blue space exposure and health outcomes, including reducing harm and building and restoring capacities.^29,31^ The first pathway refers to the mediating role of environmental hazards such as air pollution (eg, fine particulate matter) in the relationship between green space and blue space exposure and health outcomes.^29,31^ For example, some studies found that the concentrations of air pollutants seem to be lower around coastal areas due to the dispersing effect of sea breezes,^32^ while air pollutants such as fine particulate matter may damage brain structures (eg, synapses), disrupt nerve signals and lead to memory deficits.^7,10^ The second pathway involves building capacities, which means green space and blue space exposure may contribute to better health outcomes by encouraging more physical activity (PA)^29,31,33^ and promoting positive social relations.^34,35^ Also, PA may stimulate neurotransmitters and promote brain plasticity,^4,7^ and there is evidence that positive social relations may keep the brain active and build cognitive reserve,^4,7^ which both are protective factors for cognitive function.^4,7^ The last pathway is restoring capacities, which suggests that natural environments such as water bodies may help people improve their resilience to stressful events.^29,31^ For example, the ART suggests that natural environments facilitate the restoration of cognitive capacity by replenishing directed attention resources exhausted by cognitively demanding urban contexts. This process occurs through effortless engagement with nature’s ‘soft fascination’, resulting in improved attention, reduced mental fatigue and enhanced cognitive performance.^36^

In summary, through a review of the previous research on how green space and blue space influence cognitive function in later life, there are three research gaps. First, previous studies mainly focus on green space, while less attention has been paid to the health benefits of blue space exposure. Second, while much scholarly attention has been paid to the link between environmental exposures and health outcomes of the general population, such as obesity and mental health, there is surprisingly little empirical research on the link between environmental exposure and cognitive function in older adults. Third, studies focusing on green space–cognitive function and blue space–cognitive function associations remain scarce. Also, the pathways linking green space and blue space to cognitive function are still unclear.

With these research needs in mind, this study aims to examine the association between exposure to green space and blue space and cognitive function, and its potential psychosocial and environmental pathways (environmental hazards and building capacities) among an older adult population from NI. Accordingly, first, we hypothesised that there is a total positive effect of exposure to green space/blue space on cognitive function. Second, we hypothesised that different metrics of green space/blue space exposure (eg, accessibility of green space/blue space vs the availability of green space/blue space) may be associated with cognitive function through different pathways.

## Data and methods

This study used existing data from two sources to respond to the above hypotheses: the Northern Ireland Cohort for the Longitudinal Study of Ageing (NICOLA) and integrated environmental data (described in sections 2.1–2.5 below).

### Study design and population

This longitudinal study was based on data on adults >50 years derived from the NICOLA.^37^ NICOLA Wave 1 was conducted between 2014 and 2016, and Wave 2 was conducted between 2017 and 2020. Participants were selected based on multistage selection and geographical clustering of addresses. Participants’ addresses were selected from the Business Service Organisation General Practitioner Register Database, which was then merged with the Land and Property Services Pointer Database. Finally, 8478 participants aged over 50 years living in private households (those living in care homes were not included) in NI were included in Wave 1, and 6097 participants from Wave 1 agreed to participate in the second wave and were included in Wave 2. Although all the participants were invited to undergo a health assessment in the NI Clinical Research Facility at Belfast City Hospital, since attending the clinical cognition test is quite time-consuming, only a subgroup of participants took part in the cognition test and had valid information for cognitive function (Wave 1=3741, Wave 2=5928). After eliminating respondents with incomplete or missing data on exposures and covariates, we had a viable sample size of 3114 older adults in Wave 1 and 4747 in Wave 2. More details of the study design can be found in Neville *et al*.^37^ We further conducted a t-test between Mini-Mental State Examination (MMSE) scores for the final samples and MMSE scores for the excluded samples due to missing information. The result indicated that there is no significant difference between them.

### Green space and blue space measures

#### Availability of green space and blue space

The availability of green space and blue space was calculated as the proportion of green space and blue space within the 800 m circular buffer around the centroid of each participant’s address. We defined green space as vegetation, including both grassland and woodland, while blue space was defined as water bodies, including both saltwater and freshwater. The Land Cover Map 2015 (LCM2015) and 2018 (LCM2018) vector data were used for the analysis.^38^ The LCM was created by the UK Centre for Ecology and Hydrology using the classification of satellite images in different years across the UK, which can provide land use information in 21 classes.^38^

#### Normalised Difference Vegetation Index and Normalised Difference Water Index

Normalised Difference Vegetation Index (NDVI) measures the greenness of vegetation, while Normalised Difference Water Index (NDWI) measures the blueness of water bodies. We used Sentinel-2 remote sensing images provided by the European Space Agency to quantify NDVI^39^ and NDWI^40^ at 10 m × 10 m spatial resolution. The images for this study were acquired in 2015 and 2018, which were linked to the Wave 1 and Wave 2 data of the NICOLA, respectively. We chose to use an 800 m buffer instead of 1000 m (distance usually used for defining 10–15 min walking distance for the general population,^41^ as older adults have lower daily mobility than younger adults).^42^ Most of the participants from Wave 1 participated in the study in 2015, while most of the participants from Wave 2 participated in the study in 2018, so in order to simplify the operation, these 2 years were used to assess the green space and blue space measures. We averaged pixel values of NDVI and NDWI within the 800 m circular buffer around the centroid of each housing address. NDVI/NDWI values vary between −1 and 1, and a higher value indicates a higher density of healthy vegetation/water bodies, which also reflects the quality of green and blue spaces.

#### Accessibility of green space and blue space

We defined accessibility of green space and blue space as the distance in metres to the nearest public park and coastline with publicly accessible points for each participant. Green space and blue space amenities were collected from the GreenspaceNI Map (https://www.out-scape.com/news/green spaceni-map/) created by Outdoor Recreation Northern Ireland.

### Cognitive function

The outcome of interest in this study was cognitive function, which was measured using the MMSE scores.^43^ The MMSE is a well-validated means of grading global cognitive function among ageing adults.^44^ There are five domains of cognitive function (orientation, registration, attention and calculation, recall and language) assessed in the MMSE. Each question was scored 0 or 1. The final scores range from 0 to 30, and higher scores indicate better cognitive function. Norms in an Irish context indicate that a score of <25 indicates probable dementia in NI samples.^45^

### Mediators

#### Air pollution

We used particulate matter with a diameter of 2.5 µm or less (PM_2.5_) as a proxy for air pollution.^46,47^ We used the 2015 and 2018 Background Mapping data for local authorities provided by the Department for Environment, Food and Rural Affairs (DEFRA). Annual PM_2.5_ data points (μg/km^3^) are modelled based on the UK Pollution Climate Mapping approach, which is used to model the annual mean background and roadside concentrations for the UK (DEFRA, 2020). The data are presented in 1 km × 1 km grid squares of spatial resolution. We calculated the annual average PM_2.5_ concentration using the average grid values within the 1000 m circular buffer around the centroid of each housing address.

#### Building capacities

Following previous studies,^48,49^ people’s ability to build capacities is usually evaluated by PA and social capital, such as social contact and social support. As such, we used multiple measures to capture ‘building capacities’ as a mediating pathway.

Moderate-to-vigorous PA (MVPA) and walking were measured by the International Physical Activity Questionnaire-Short Form, which assesses PA information in a typical week.^50^ The Global Physical Activity Questionnaire has been validated for its reliability in the NI population.^51^ MVPA was calculated by combining both total minutes of vigorous PA per week and total minutes of moderate PA per week (minutes). Also, the total minutes spent walking per week (minutes) was included as another proxy of PA.

Following existing studies,^52^ we used a single self-reported question to quantify social contacts within the neighbourhood. Respondents were asked, ‘How often do you speak to neighbours?’. The answers range from ‘never’ (1) to ‘on most days’ (5). Higher values meant more frequent social contact.

We used a single self-reported question to quantify social support following existing studies.^53^ Respondents were asked, ‘If you had a serious personal crisis, how many people, if any, do you feel you could turn to for comfort and support?’. The total number of people was calculated and treated as a proxy of social support. Higher numbers meant a higher level of social support.

### Covariates

The NICOLA survey includes information on demographic, socio-economic and other individual data from participants. Following previous studies,^29,54^ we controlled for sex, age, marital status, education, employment, smoking and drinking behaviour, the presence of cardiovascular diseases (CVDs) including hypertension, area level Multiple Deprivation Measure,^55^ region^56^ and duration of residency (in years). We also used the Brief Resilience Scale (BRS)^57,58^ to calculate participants’ resilience as a covariate. BRS has six questions (online supplemental table S1), and respondents were asked to indicate the extent to which they agree or disagree with each of the statements. A five-point Likert-type scale ranging from ‘strongly disagree’ (1) to ‘strongly agree’ (5) was used. We used the average score of all items to measure the level of resilience (Cronbach’s alpha >0.90). Higher BRS scores mean a higher level of resilience. Then, we draw a directed acyclic graph (online supplemental figure S1)^59^ to present the association among exposure, mediators, outcome and covariates, and the detailed links among different variables were presented in the supplement file. Summary statistics of variables are presented in table 1.

**Table 1:** Selected characteristics of the study population for the NICOLA Wave 1 sample (n=3043)

Variable		Number/value	%/SD
Sex	Female	1588	52.19
	Male	1455	47.81
Age	<60	1173	38.55
	60–69	1104	36.28
	70–79	595	19.55
	>80	171	5.62
Marital status	Married/cohabiting/same-sex civil partnership	2273	74.70
	Never married	173	5.69
	Separated/divorced/widowed	597	19.62
Education	Primary/none	398	13.08
	Secondary	1364	44.82
	Higher	1281	42.10
Employment	Retired	1501	49.33
	Employed	1253	41.18
	Unemployed	71	2.33
	Others	218	7.16
Area-level Multiple Deprivation Measure	1 Most deprived	395	12.98
	2	525	17.25
	3	612	20.11
	4	644	21.16
	5 Least deprived	867	28.49
Region	Urban	526	17.29
	Intermediate	1721	56.56
	Rural	796	26.16
Duration of residency	<5 years	143	4.70
	≥5 years	2900	95.30
The presence of CVDs	Yes	1601	52.61
	No	1442	47.39
Current smoker	Yes	517	16.99
	No	2526	83.01
Current drinker	Yes	2110	69.34
	No	933	30.66
BRS scores	BRS scores	3.59	0.77
Cognitive function	MMSE Scores	29.59	8.81
Green space	NDVI	0.36	0.17
	Availability of green space: the proportion of green space	0.41	0.32
	Accessibility of green space: distance to the nearest green space (m)	3237.59	4120.28
Blue space	NDWI	−0.26	0.16
	Availability of blue space: the proportion of blue space	0.01	0.04
	Accessibility of blue space: distance to the nearest blue space (m)	18 345.74	14 904.48
Mediators	MVPA (min/per week)	228.29	419.15
	Walking (min/per week)	234.61	313.74
	Social contact	4.00	0.96
	Social support (persons)	6.87	4.26
	PM_2.5_ (μg/km^3^)	6.18	1.05

BRS, Brief Resilience Scale; CVDs, cardiovascular diseases; MMSE, Mini-Mental State Examination; MVPA, moderate-to-vigorous intensity physical activity; NDVI, Normalised Difference Vegetation Index; NDWI, Normalised Difference Water Index; NICOLA, Northern Ireland Cohort for the Longitudinal Study of Ageing; PM 2.5, fine particles that have a diameter less than 2.5 µm.

### Statistical analysis

Since there are no latent variables in this study and to test the parallel mediating effects of all the mediators at the same time, we used structural equation models (SEMs)^60^ with only observed variables to test our hypotheses and model the total effect of blue space on cognitive function as well as the potential mediating pathways. There was no multicollinearity issue among the variables in this model, based on the variance inflation factor (<5) values. Since there are two waves of data, the measures of MMSE scores across two waves were clustered within each individual, and individuals were clustered within different electoral wards. As such, we used the multilevel SEM with random intercepts, which allows intercepts to vary among individuals and wards.^61^ Such an operation combines the SEMs with only observed variables and multilevel models, which is suitable for the longitudinal study design.

To simplify the calculation, we used the parallel mediation model (figure 1), which assumes that mediators are not related and there is no interaction effect between the exposure and mediators on the outcome.^62^ We tested each of the green space and blue space exposure metrics separately to avoid potential multicollinearity, so there are three SEMs. The goodness of fit in the normal SEMs was evaluated by cut-offs of comparative fit index (CFI >0.90), root mean square error of approximation (RMSEA <0.05) and standardised root mean square residual (SRMSR <0.05) suggested by Hu and Bentler.^63^ Finally, we calculated the direct effect, indirect effect and the proportion of effect size for each of the mediators in the final models following Preacher and Hayes.^64^ For sensitivity analysis, we repeated our analyses using 600-metre and 1000-metre buffers for assessing the green space and blue space metrics, but the results remained similar (online supplemental figures S2-S5). Therefore, we only presented the results of the 800-metre buffer in the main text. Stata V.15.1 was used for the statistical analysis (Stata, College Station, Texas, USA). All models were conducted with a complete case analysis.

**Figure 1:**
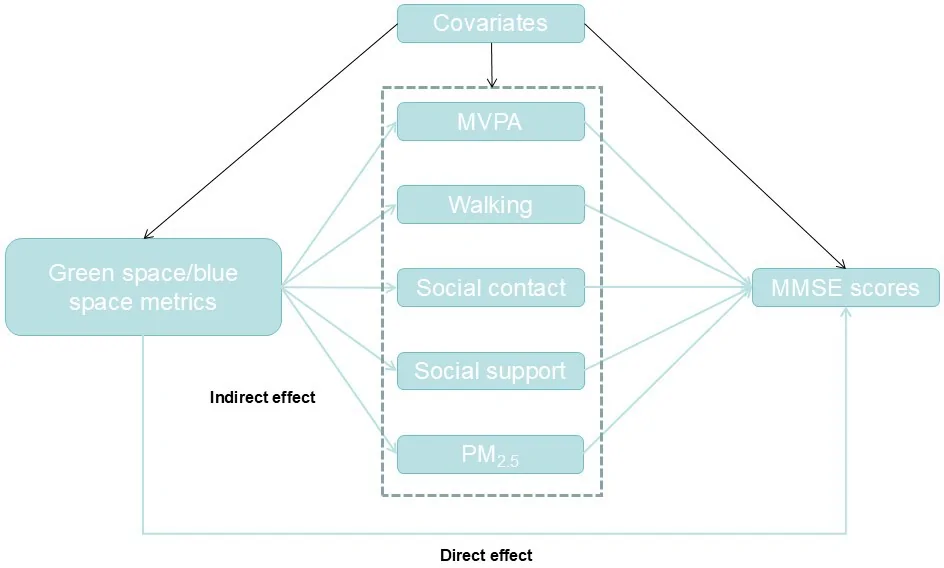
The parallel mediation models of this study (green space/blue space metrics include the availability of green space/blue space, accessibility of green space/blue space and NDVI/NDWI. Their effects are tested separately in three different models). MMSE, Mini-Mental State Examination; MVPA, moderate-to-vigorous physical activity; NDVI, Normalised Difference Vegetation Index; NDWI, Normalised Difference Water Index; PM_2.5_, particulate matter with a diameter of 2.5 µm or less.

## Results

### Characteristics of the baseline study population

Table 1 summarises the characteristics of the baseline study population. The mean MMSE score of the total participants was 29.59 (SD=8.81) for the baseline population, while the mean MMSE score was 28.41 (SD=2.05) for the population in Wave 2. This is well above the cut-off for dementia, indicating that the sample had relatively preserved cognitive function. The average value of NDVI was 0.36 (SD=0.17), while the average value for the proportion of green space and distance to the nearest green space was 0.41 (SD=0.32) and 3237.59 (SD=4120.28) m, respectively. The average value of NDWI was −0.26 (SD=0.16), while the average value for the proportion of blue space and distance to the nearest blue space was 0.01 (SD=0.04) and 18 345.74 (SD=14 904.48) m, respectively. As for mediators, the average MVPA and walking duration were 228.29 (SD=419.15) and 234.61 (SD=313.74) min/week, respectively. Also, the mean social contact and social support were 4.00 (SD=0.96) and 6.87 persons (SD=4.26), respectively. The average PM_2.5_ was 6.18 μg/km^3^ (SD=1.05), which is close to the 2021 WHO air quality guidelines (5 μg/km^3^).

The mean age was 63.30 years, and 47.81% of the participants were male. About one-third of the participants were married/cohabiting/same-sex civil partnership (74.70%), and only 13.08% had primary/none qualifications. Nearly 49.33% of the participants were retired, and 95.30% had resided at the current address for more than 5 years. Hence, 12.98% of the participants lived in the most deprived areas, and 17.29% lived in the urban regions. Last, nearly half of the participants (52.61%) had CVDs, 16.99% were current smokers and 69.34% were current drinkers.

### The results of parallel mediation models for SEMs

We obtained acceptable parallel mediation models for all green space and blue space metrics (figures 2 and 3): SRMSR <0.035, RMSEA <0.035, CFI >0.900.

**Figure 2:**
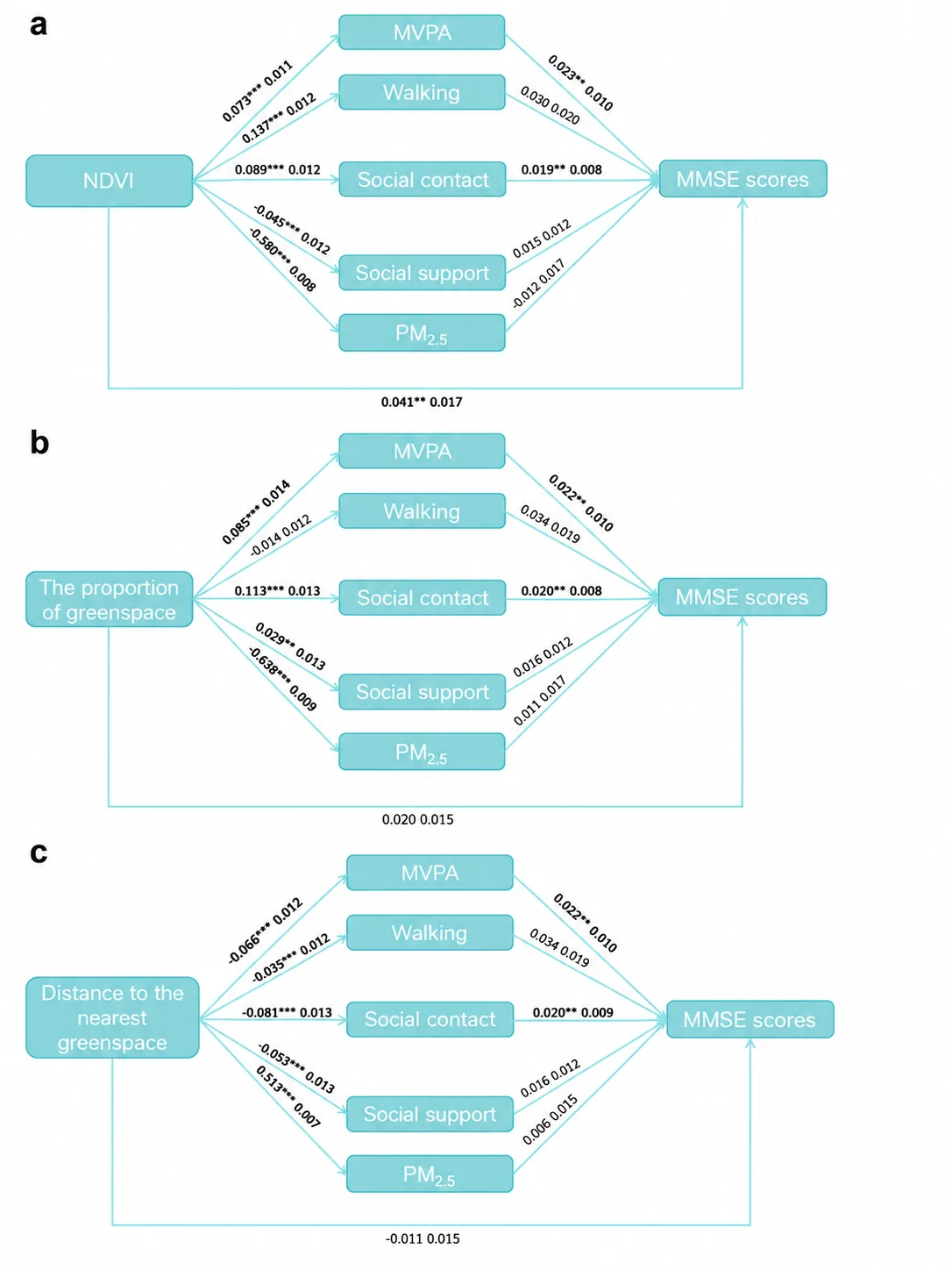
Reports results for the parallel mediation model in the SEM for green space metrics, and the effect sizes were presented as the standardised coefficients. (**a**) NDVI was positively associated with MVPA (Coef.=0.073; SE=0.011), walking (Coef.=0.137; SE=0.012) and social contact (Coef.=0.089; SE=0.012) but negatively associated with social support (Coef.=−0.045; SE=0.012) and PM_2.5_ (Coef.=−0.580; SE=0.008). (**b**) The proportion of green space was positively associated with MVPA (Coef.=0.085; SE=0.014), social contact (Coef.=0.113; SE=0.013) and social support (Coef.=0.029; SE=0.013) but negatively associated with PM_2.5_ (Coef.=−0.638; SE=0.008). (**c**) Distance to the nearest green space was negatively associated with MVPA (Coef.=−0.066; SE=0.012), walking (Coef.=−0.035; SE=0.012), social contact (Coef.=−0.081; SE=0.013) and social support (Coef.=−0.053; SE=0.013) but positively associated with PM_2.5_ (Coef.=0.513; SE=0.007). Also, MVPA and social contact were positively related to MMSE scores, and there is no evidence that the rest of the mediators were associated with MMSE scores. MMSE, Mini-Mental State Examination; MVPA, moderate-to-vigorous physical activity; NDVI, Normalised Difference Vegetation Index; PM_2.5_, particulate matter with a diameter of 2.5 µm or less; SEM, structural equation model.

**Figure 3:**
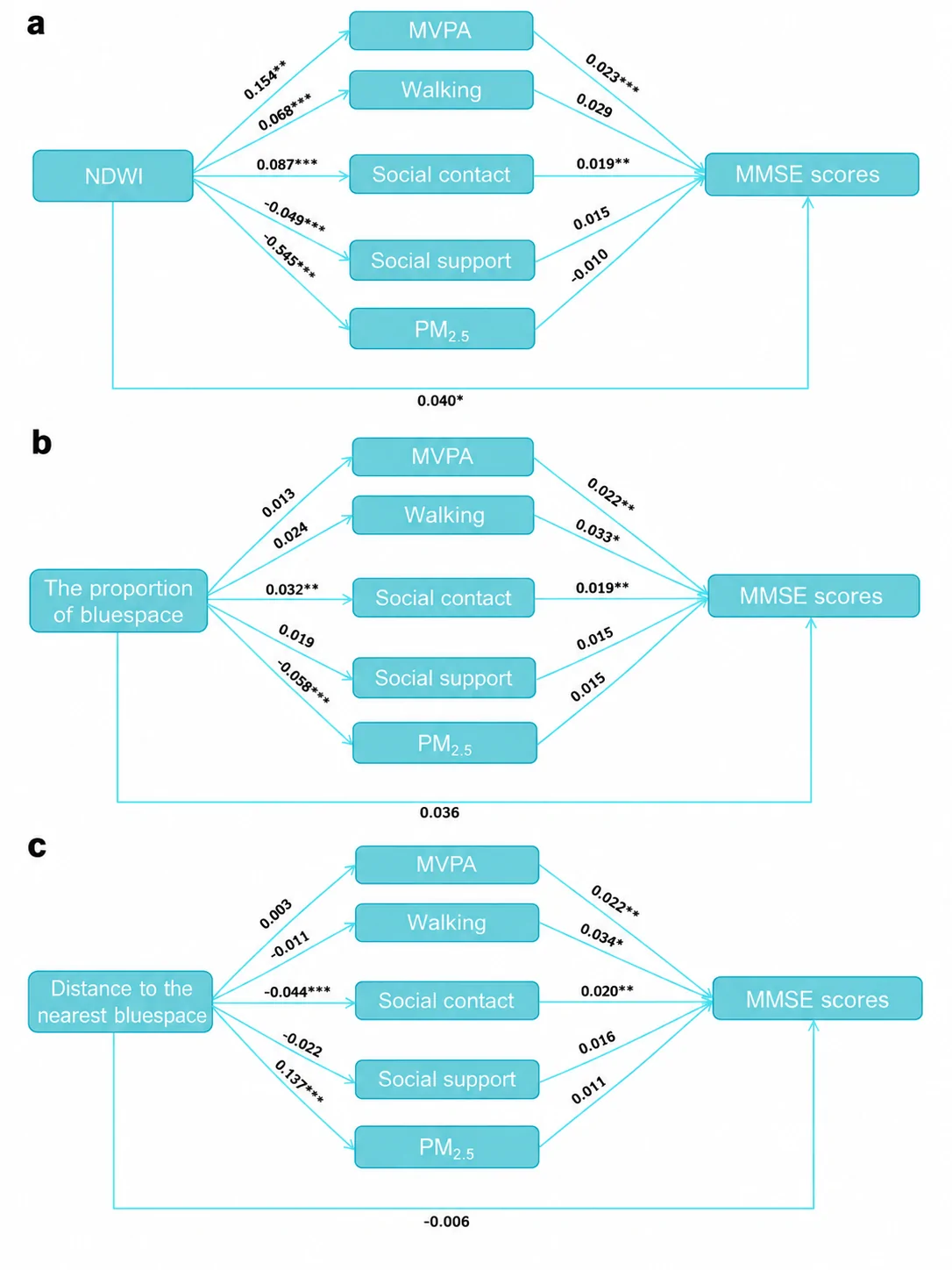
Reports results for the parallel mediation model in the SEM for blue space metrics, and the effect sizes were presented as the standardised coefficients. (**a**) NDWI was positively associated with MVPA (Coef.=0.154; SE=0.012), walking (Coef.=0.069; SE=0.011), social contact (Coef.=0.087; SE=0.012) and social support (Coef.=0.049; SE=0.012) but negatively associated with PM_2.5_ (Coef.=−0.545; SE=0.008). (**b**) The proportion of blue space was positively associated with social contact (Coef.=0.032; SE=0.015) but negatively associated with PM_2.5_ (Coef.=−0.058; SE=0.011). (**c**) Distance to the nearest blue space was negatively associated with social contact (Coef.=−0.044; SE=0.013) but positively related to PM_2.5_ (Coef.=0.157; SE=0.011). Also, MVPA and social contact were positively related to MMSE scores, and there is no evidence that the rest of the mediators were associated with MMSE scores. MMSE, Mini-Mental State Examination; MVPA, moderate-to-vigorous physical activity; NDWI, Normalised Difference Water Index; PM_2.5_, particulate matter with a diameter of 2.5 µm or less; SEM, structural equation model.

Table 2 indicates that the standardised effect of NDVI on MMSE scores through MVPA is 0.0017 (SE=0.0008; %Mediated=3). Also, there was evidence that there is a direct association between NDVI and MMSE scores (effect size: Coef.=0.041; SE=0.017). The standardised effect of the proportion of green space on MMSE scores through social contact is 0.0023 (SE=0.0009; %Mediated=13). However, there was no evidence to suggest that there is a direct association between the proportion of green space and MMSE scores. The standardised effect of distance to the nearest green space on MMSE scores through MVPA is −0.0015 (SE=0.0007; %Mediated=7). However, there was no evidence to suggest that there is a direct association between distance to the nearest green space and MMSE scores.

**Table 2:** Direct and indirect effects of green space and blue space indicators on MMSE scores

Predictors	Mediators	Direct effect	Indirect effect	%Mediated	R-squared*
Distance to the nearest green space	MVPA	−0.011 (0.015)	−0.0015** (0.0007)	7	0.056
	Walking		−0.0012 (0.0007)	NA	0.037
	Social contact		−0.0016* (0.0008)	NA	0.035
	Social support		−0.0008 (0.0006)	NA	0.016
	PM_2.5_		−0.0034 (0.0084)	NA	0.405
The proportion of green space	MVPA	0.020 (0.015)	0.0019* (0.0009)	NA	0.058
	Walking		−0.0005 (0.0004)	NA	0.035
	Social contact		0.0023** (0.0009)	13	0.040
	Social support		0.0005 (0.0004)	NA	0.014
	PM_2.5_		−0.0070 (0.0108)	NA	0.479
NDVI	MVPA	0.041** (0.017)	0.0017** (0.0008)	3	0.056
	Walking		0.0041 (0.0027)	NA	0.053
	Social contact		0.0017* (0.0008)	NA	0.036
	Social support		−0.0007 (0.0005)	NA	0.015
	PM_2.5_		0.0070 (0.0098)	NA	0.420
Distance to the nearest blue space	MVPA	−0.006 (0.011)	0.0001 (0.0002)	NA	0.051
	Walking		−0.0004 (0.0004)	NA	0.035
	Social contact		−0.0009** (0.0004)	10	0.029
	Social support		−0.0004 (0.0003)	NA	0.015
	PM_2.5_		−0.0017 (0.0018)	NA	0.132
The proportion of blue space	MVPA	0.036 (0.030)	0.0003 (0.0002)	NA	0.052
	Walking		0.0008 (0.0006)	NA	0.036
	Social contact		0.0006 (0.0004)	NA	0.029
	Social support		0.0003 (0.0002)	NA	0.015
	PM_2.5_		−0.0009 (0.0007)	NA	0.112
NDWI	MVPA	0.040*(0.025)	0.0035** (0.0015)	8	0.056
	Walking		0.0020 (0.0014)	NA	0.057
	Social contact		0.0017* (0.0008)	NA	0.036
	Social support		0.0007 (0.0006)	NA	0.016
	PM_2.5_		−0.0055 (0.0081)	NA	0.383

*p<0.10, **p<0.05.

* The R-squared is for the normal SEMs rather than the multilevel SEM.

MMSE, Mini-Mental State Examination; MVPA, moderate-to-vigorous intensity physical activity; NA, not applicable; NDVI, Normalised Difference Vegetation Index; NDWI, Normalised Difference Water Index; PM_2.5_, fine particles that have a diameter less than 2.5 µm; SEM, structural equation model.

Table 2 indicates that the standardised effect of NDWI on MMSE scores through MVPA is 0.0035 (SE=0.0015; %Mediated=8). However, there is no evidence to suggest that there is a direct association between NDWI and MMSE scores. There is no evidence to suggest that there is a direct or indirect association between the proportion of blue space and MMSE scores. The standardised effect of distance to the nearest blue space on MMSE scores through social contact is −0.0009 (SE=0.0004; %Mediated=10), but there was no evidence to suggest that there is a direct association between distance to the nearest blue space and MMSE scores.

## Discussion

This study extends previous research on the association between green space and blue space exposure and cognitive function. Specifically, it enhances our understanding of the different pathways through which green space and blue space exposure influence cognitive function in later life. The key finding in this study is that MVPA and social contact mediate the association between green space/blue space metrics and MMSE scores.

### The mediating role of physical activity

The results show that MVPA is one of the significant mediators. Green and blue spaces may encourage residents living nearby to engage in outdoor PA since they provide an accessible and enjoyable setting for MVPA.^65^ Moreover, scientific evidence shows that MVPA in a natural environment may generate more benefits than in other environments.^66^ Also, MVPA has long been a positive factor for cognitive function, since it can promote energy levels through the release of hormones (eg, endorphins) and delivery of oxygen throughout the body.^67^ However, we also found no evidence that MVPA mediated the association between the proportion of blue space and MMSE scores in older adults. A possible explanation is that some water bodies near the participants may not be of good quality, which can discourage their use of them and their willingness to conduct PA near them.^68^ This also may be because the large blue space amenities (eg, lakes) may not be close enough for participants in this study to encourage frequent MVPA.^69^

### The mediating role of social contact

Another important finding in this study is that social contact also mediated the association between green space and blue space exposure and cognitive function in later life. Green and blue spaces can provide a comfortable open space for people to meet up and socialise with their friends, neighbours and family members.^70^ Hence, more frequent social contact among neighbours increases the level of neighbourhood social cohesion, which contributes to better cognitive function by facilitating collective efficacy and discouraging unhealthy lifestyles due to the spread of more health-related information.^71^ However, there is no evidence that social contact mediated the NDWI/NDVI—MMSE scores, the proportion of blue space—MMSE scores or the accessibility of green space—MMSE scores associations. The unknown quality of green and blue spaces may again explain the above associations.^34^

### The direct effect of green space

We also found a positive and direct effect of NDVI on MMSE scores in older adults. This is consistent with previous studies in different countries or regions such as Australia,^72^ USA,^73,74^ UK,^75^ China,^76^ Spain,^77^ Bulgaria,^78^ Germany^79^ and South Korea,^80^ which also found a positive association between green space exposure and cognitive function in older adults. However, two studies in the USA^81^ and Taiwan^82^ suggested there is no evidence that green space exposure is associated with cognitive function in older adults. One of the explanations for our results may be that green space exposure may be able to result in direct and momentary psycho-physiological responses through visual exposure.^27^ Another explanation is that we may have neglected some potential mediators, which may overestimate the direct effect of green space metrics on MMSE scores. For example, we did not include some potential mediators, such as cognition-related biomarkers^83^ and biodiversity metrics^84^ due to the unavailability of data. Although the effect of mediators such as walking, social support and PM_2.5_ did not achieve a significance level, there is no conclusive evidence that their mediating roles do not exist in the association between green space exposure and cognitive function for older adults. There are several explanations for such a result. First, some of the mediators are self-reported, so they can have measurement bias and may not be able to reflect the actual level of the proposed mediators. Also, all exposure metrics in this study are only proxies and may not be able to reflect participants’ use of green and blue spaces accurately. Second, we mainly measured social support based on participants’ general perceived support, which not only includes support from neighbours but also includes support from friends or relatives living far away. Therefore, such a variable may not necessarily be related to the surrounding outdoor environments of participants’ housing addresses.

### Strengths and limitations

Several strengths need to be emphasised. First, different from earlier studies using data collected in a single city, the present study used data collected from a representative national cohort in NI, which is large in sample size and heterogeneous in environmental contexts. Second, we used both Wave 1 and Wave 2 of the NICOLA, which makes this study a longitudinal study and can provide us with stronger scientific evidence on the relationship between green space and blue space exposure and cognitive function in later life. Third, we used MMSE scores to evaluate cognitive function rather than self-reported questions, which is a more solid and reliable proxy of cognitive function. Fourth, the green space and blue space exposures were assessed based on the housing addresses rather than the residential neighbourhood, which ensures we get more precise metrics of green space and blue space exposure. Last, we have included different metrics of green space and blue space exposure, which makes our analysis more comprehensive.

Our study is also not without limitations. First, we only have two waves of NICOLA data, which may not be enough to reflect significant changes in cognitive function over time, since mediation analysis usually requires a minimum of three waves of data to enable a high level of causal inference.^85^ Second, some of the mediators are self-reported and may still have bias. Third, we only included metrics of green space and blue space quantity, while quality was neglected due to the availability of data.^86^ Therefore, future studies should try to focus more on the effect of green space and blue space quality. Fourth, some of our mediators are self-reported, which may be influenced by recall and measurement bias.^87^ Also, some of the important mediators, such as biomarkers (eg, brain-derived neurotrophic factor, which may be related to both cognitive function and older adults’ physical mobility in the outdoor environment), were not included and should receive more attention in future research. Last, there may be synergy or interference effects among different mediators since we used multivariate mediation analysis.

## Conclusion

To our knowledge, this study is the first to explore associations between neighbourhood blue space exposure and cognitive function in later life using different exposure assessment strategies. The green and blue space exposure influences cognitive function mainly through MVPA and social contact. Our findings suggested that green space and blue space exposure are important predictors of cognitive function in later life and that they may exert their effect via multiple pathways. Green space and blue space exposure may contribute to healthy ageing.

## Supplementary material

10.1136/bmjph-2025-003080 online supplemental file 1

## Data Availability

Data may be obtained from a third party and are not publicly available.
