# Supplementary material for: Exploring the pathways between green space and blue space, and cognitive function in later life: a mediation analysis in Northern Ireland
Source: BMJ Public Health. 2026 Oct 7;4(4):e003080. doi: 10.1136/bmjph-2025-003080 (PMC13646930; doi:10.1136/bmjph-2025-003080)
Supplement: Supplementary file 1 [file bmjph-4-4-s001.doc]

Supplement file

Table S1: The Brief Resilience Scale

| Number | Items | Response |
| --- | --- | --- |
| 1 | I tend to bounce back quickly after hard times. | {1, Strongly disagree} {2, Disagree} {3, Neutral} {4, Agree} {5, Strongly agree} |
| 2 | I have a hard time making it through stressful events (reversed coding). | {1, Strongly disagree} {2, Disagree} {3, Neutral} {4, Agree} {5, Strongly agree} |
| 3 | It does not take me long to recover from a stressful event. | {1, Strongly disagree} {2, Disagree} {3, Neutral} {4, Agree} {5, Strongly agree} |
| 4 | It is hard for me to snap back when something bad happens (reversed coding). | {1, Strongly disagree} {2, Disagree} {3, Neutral} {4, Agree} {5, Strongly agree} |
| 5 | I usually come through difficult times with little trouble. | {1, Strongly disagree} {2, Disagree} {3, Neutral} {4, Agree} {5, Strongly agree} |
| 6 | I tend to take a long time to get over set-backs in my life (reversed coding). | {1, Strongly disagree} {2, Disagree} {3, Neutral} {4, Agree} {5, Strongly agree} |

Fig S1 Directed acyclic graph for the association between blue space exposure and MMSE scores, created with the help of dagitty.net. First, the core elements in the model are exposure (UGBS), outcome (MMSE scores), and mediators (PM_2.5_, social support, social contact, walking, and MVPA). The exposure is linked to all mediators and the outcome, while all mediators are also linked to the outcome. Second, another set of elements are the core covariates (red circles at the top right corner), which are linked to exposure, outcome, and mediators. Third, the duration of residency is also a covariate, but it is only linked to individual-level mediators (social support, social contact, and MVPA) and the outcome. Fourth, although CVD is also considered a potential mediator, the SEM model does not accurately calculate the mediation effect of a binary variable. Also, we mainly focused on the behavioural and environmental mediators in this study. Therefore, we controlled for CVD but did not calculate its mediation effect. Last, BRS scores, smoking and drinking were only considered as the risk factors of the outcome, so they are only linked to the outcome.

Fig. S2. SEM for the association between green space and MMSE scores (consistent mediation effect; buffer size = 600-m). Note: Significance levels: * p < 0.05, ** p < 0.01, *** p < 0.001. The model was adjusted for all covariates shown in Table 1. All coefficients are standardised. All variables were collected in both waves 1 and 2.

Fig. S3. SEM for the association between green space and MMSE scores (consistent mediation effect; buffer size = 1000-m). Note: Significance levels: * p < 0.05, ** p < 0.01, *** p < 0.001. The model was adjusted for all covariates shown in Table 1. All coefficients are standardised. All variables were collected in both waves 1 and 2.

Fig. S4. SEM for the association between blue space and MMSE scores (consistent mediation effect; buffer size = 600-m). Note: Significance levels: * p < 0.05, ** p < 0.01, *** p < 0.001. The model was adjusted for all covariates shown in Table 1. All coefficients are standardised. All variables were collected in both waves 1 and 2.

Fig. S5. SEM for the association between blue space and MMSE scores (consistent mediation effect; buffer size = 1000-m). Note: Significance levels: * p < 0.05, ** p < 0.01, *** p < 0.001. The model was adjusted for all covariates shown in Table 1. All coefficients are standardised. All variables were collected in both waves 1 and 2.
